# Use of Sleeve Nets to Improve Survival of the Boisduval Silkworm, *Anaphe panda*, in the Kakamega Forest of Western Kenya

**DOI:** 10.1673/031.010.0601

**Published:** 2010-02-25

**Authors:** N. Mbahin, S. K. Raina, E. N. Kioko, J. M. Mueke

**Affiliations:** ^1^ Commercial Insects Programme, ICIPE-African Insect Science for Food and Health, P. O. Box: 30772-00100 Nairobi, Kenya; ^2^ Department of Zoological Sciences, Kenyatta University, P. O. Box: 43844, Nairobi, Kenya

**Keywords:** conservation, exclusion, mortality, silk farming, silkmoth

## Abstract

Prospects for development of a wild silk industry in Africa would be improved if silkworm survival during mass production could be improved. A study on the survival of the Boisduval silkworm, *Anaphe panda* (Boisduval) (Lepidoptera: Thaumetopoeidae) was conducted with and without protection by net sleeves in two different forest habitats (natural and modified) in the Kakamega forest of western Kenya. Overall, cohort survival was significantly higher (*P* < 0.001) in the natural than in the modified forest, but larval survival was improved over three-fold by protection with net sleeves in both habitat types. In the modified forest, only 16.8% of unprotected larvae survived to the pupal stage and formed cocoons, whereas 62.3% survived in the same environment when they were protected with net sleeves. In the natural forest, 20.4% of unprotected larvae survived, whereas 67.7% survived in net sleeves. There was also a significant effect of season; cohorts of larvae that eclosed in the wet season had significantly lower survival than those eclosing in the dry season (P = 0.02). Sources of mortality appeared to be natural enemies (parasites, predators and diseases) and climatic factors.

## Introduction

In many parts of the developing world, people seek diversified sources of income, especially those that are sustainable and environmental friendly. Due to current population growth in Kenya, pressure on the Kakamega forest is growing because the forest plays an important role in satisfying the daily needs and income of local people. Wild silk farming is a unique, ecologically friendly industry with a great potential for employment generation, artisanal development and export earnings (Kioko et al. 1999). African species of silkmoths provide strong silk of high commercial value (Raina 2004). The Boisduval silkworm, *Anaphe panda* (Boisduval) (Lepidoptera: Thaumetopoeidae) shows the best potential for wild silk production since it produces a huge cocoon that is communally weaved by 20–200 silkworm larvae. Wild silk farming is among the industries that might assist resource-poor farmers of this region to escape a vicious cycle of poverty, while providing an incentive for forest conservation (Kioko et al. 2000). In countries where rural communities depend on subsistence farming, wild silkmoth cultivation can be a supplementary activity for income generation while simultaneously conserving biodiversity.

Silk has been used for textiles for thousands of years (Raina 2004). In Africa, most of the wild silkmoths belong to the families Saturniidae, Lasiocampidae, and Thaumetopoeidae. *Anaphe* species are widely distributed in the intertropical regions of continental Africa such as Nigeria, Uganda, Kenya, Cameroon, Congo and Togo. The important species used in the production of *Anaphe* silk are *Anaphe infracta* Walsingham, *Anaphe venata* Butler, *Anaphe panda* Boisduval, *Anaphe reticulata* Walker, *Anaphe carteri* Walsingham, *Anaphe moloneyi* Druce and *Anaphe ambrizia* Butler. *Anaphe* are polyphagous moths but *Bridelia micrantha* (Hochst) (Malpighiales: Euphorbiaceae) is the preferred host plant of *A. panda* in the Kakamega Forest. In nature, *B. micrantha* can be found scattered over a large area of the Kakamega forest and it flourishes in both natural tracts and those mixed with exotic species. With very little care it can be raised from seed or from cuttings that propagate rapidly and are ready for use as host plants in about a year (Gowdey 1953; Jolly et al. 1979). In the Kakamega Forest, *A. panda* silkmoths lay egg clusters under the leaves of *B. micrantha*.

The potential of the African indigenous silkmoth species for wild silk production has been well documented in Nigeria (Ashiru 1991), Uganda (Kato 2000) and Kenya (Kioko et al. 2000; Mbahin et al. 2008). However, the wild silk industry in Kenya will not be commercially viable unless technologies are developed to reduce silkworm mortality due to natural enemies and help farmers develop sustainable mass production of *A. panda* cocoons. Some studies have recommended the use of sleeve nets to protect young larvae (Kioko et al. 1999; Raina 2000; 2004; Ngoka 2003), but there are no reliable data available on the benefit of using sleeve nets to improve *Anaphe* silkworm survival in the field. The present study was conducted in two forest habitats to test whether the survival rate of *A. panda* silkworms can be improved by protection with sleeve nets.

## Materials and Methods

### Study sites

**Figure 1:**
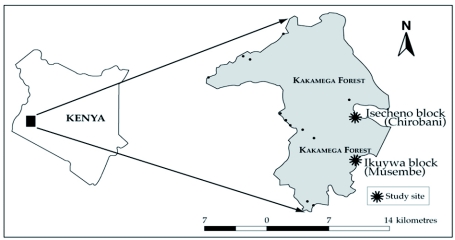
Study sites in the Kakamega Forest of western Kenya. High quality figures are available online.

The Kakamega Forest is a tropical rainforest that covers a total area of approximately 265 km^2^ and is located between latitudes 0° 10′ and 0° 21′ North and longitudes 34° 47′ and 34° 58′ East, respectively (Figure 1). It comprises several separate blocks of forest of which Isecheno (415 ha) belongs to the Lunyu sub-location whereas Ikuywa (380 ha) belongs to the Ikuywa sub-location. Two sites were used for the study: Musembe village, located in Ikuywa (a natural forest comprised exclusively of indigenous species), and Chirobani village, located in Isecheno (a modified indigenous forest comprised of a combination of native and exotic species). The exotic species are mainly pines, *Acacia* spp. (Fabaceae) and *Eucalyptus* spp. (Myrtaceae) (Mbahin et al. 2008). Indigenous plants comprise about 150 species of woody trees, 90 species of dicotyledonous herbs, 80 species of monocotyledonous herbs of which about 60 are orchids, and a further 62 species of ferns, totalling to about 380 identified species of vascular plants (KIFCON 1994).

### Environmental data

A digital hygrothermometer (Zheda Electric Apparatus Inc., http://www.zjlab.com) was used for recording daily temperature (maximum and minimum) and measurements of relative humidity were recorded four times daily (6 am; 12 am; 3 pm and 9 pm) at both sites throughout the period of study. A rain gauge was used for recording rainfall data.

### Silkworm survival

**Figure 2:**
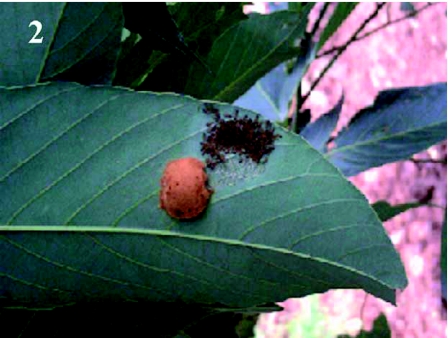
Eclosing egg mass of *Anaphe panda*. High quality figures are available online.

**Figure 3:**
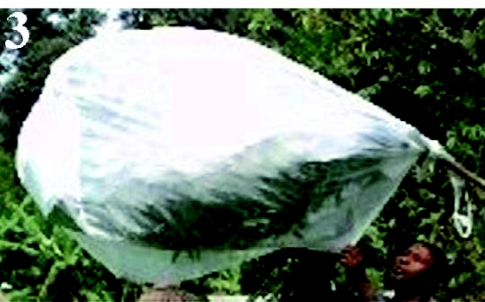
Branch of *Bridelia micrantha* with net sleeve used to enclose developing cohorts of *Anaphe panda* silkworms. High quality figures are available online.

**Figure 4:**
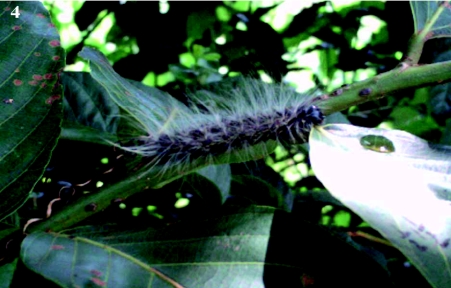
7^th^ instar larvaof *Anaphe panda* on its host plant, *Bridelia micrantha*. High quality figures are available online.

Two naturally-laid egg clusters of *A. panda* were selected on each of 150 *B. micrantha* trees that had canopies of about 10 cubic feet (Figure 2). Any additional egg clusters were removed. Each tree was divided into two experimental areas: one with a net sleeve measuring 1.5 × 1.5 × 2 m covering one cohort of larvae and a control cohort of larvae that were not covered. The net sleeves were tied closed on the branches of the host plant and a 2 m long zippered aperture \ permitted access for purpose of observation and data collection (Figure 3). Branches were selected among those that bore sufficient leaves to provide adequate food. Of 300 clusters of eggs selected, eggs hatched in a total of 221 that were included in the experiment (105 protected, 116 exposed). Observations on the possible causes of mortality were made twice weekly and counts of the surviving larvae in net sleeves and on control branches from June 2005 to June 2007. Protected cohorts were moved to new branches of the same tree two or three times during the course of development as required to ensure an adequate food supply. Only cohorts that completed all seven larval instars with some survivors (Figure 4) were included in the analysis. The mortality rate (by instar) was calculated as follows:

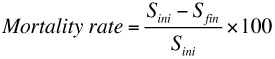

With: *S _ini_* = Number of larvae alive at the beginning of the instar and *S _fin_* = Number of larvae alive at the end of the instar.

The instantaneous death rate is the boundary of the expression below when Δ*t* → 0.

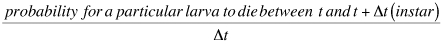

The ‘force of mortality’ (incidence rate) for each instar can be calculated as follows:

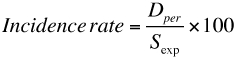

Where *D _per_* = Number of dead larvae in the specific instar and *S _exp_* = Number of surviving larvae exposed to risk in that instar.

### Data analysis

The data on numbers of larvae surviving to form cocoons were analyzed by factorial ANOVA (SAS Institute, 2003) with ‘year’ (2005/2006/2007), ‘treatment’ (protected/unprotected), forest (natural/modified), and ‘brood’ (1^st^/2^nd^) as independent variables. Analysis of survival-time data was carried out with the software Stata7 (STATACORP, 2004) and a Chi-Square test was used to compare overall cohort survival between forest types.

**Figure 5:**
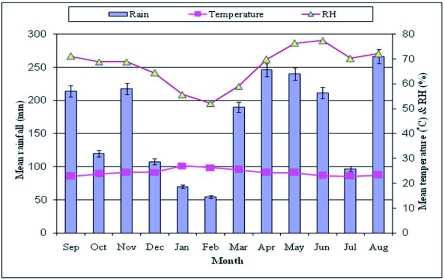
Rainfall, temperature and relative humidity in the Isecheno modified forest (2005–2007). High quality figures are available online.

**Figure 6:**
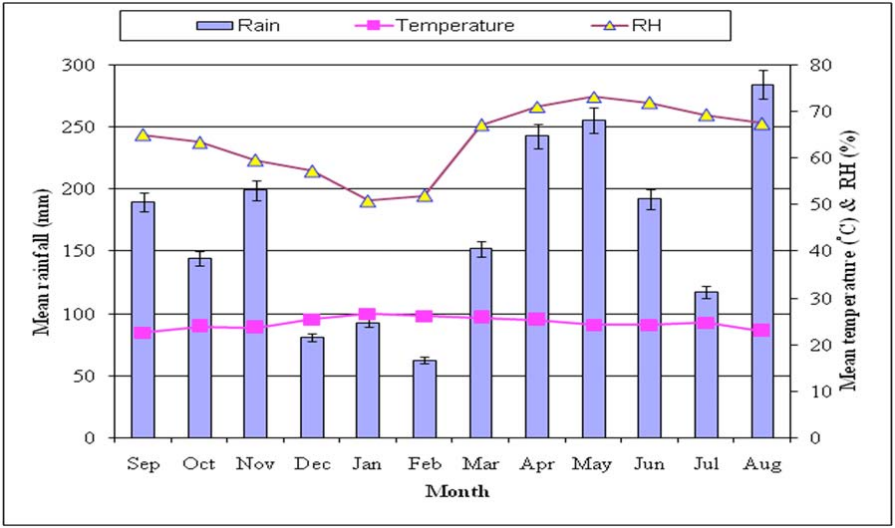
Rainfall, temperature and relative humidity in the Ikuywa natural forest (2005–2007). High quality figures are available online.

## Results

### Environmental data

Data on mean monthly average temperature, relative humidity, and rainfall for the two study sites are reported in Figures 5 and 6, respectively. Note that rainfall is bimodal in the Kakamega Forest, with a period of “long rains” from April through June, and “short rains” in August through November. During the study, the annual rainfall ranged from 180.9 to 265.9 cm in the modified forest (lsecheno), and from 188.6 to 224.7 cm in the natural forest (Ikuywa). The annual number of rainy days ranged from 196 – 219, and from 207 – 209 in the modified and natural forests, respectively. Mean monthly maximum temperature ranged from 15.5° C to 36.8° C at Isecheno, and from 16.5 – 35.6° C at Ikuywa. Mean monthly maximum humidity ranged from 45.4 – 86.2 % in the modified forest and from 35.6 – 80.9 % in the natural forest.

### Larval survival

There was no effect of ‘year’ on the number of larvae pupating (*F* = 0.09; df = 2,177; *P* = 0.910) so data were pooled across years for further analysis. In both forest types, mortality rates tended to be higher for young larvae (1^st^ to 4^th^ instar) than for older instars (5^th^ to 7^th^) (Table 1), but the main effect of forest type was not significant (*F* = 2.49; df = 1, 178; *P* = 0.117). However, the effect of the sleeve net protection treatment was highly significant (*F* = 229.26; df = 1,178; *P* < 0.001) and cohorts eclosing in the wet season (2^nd^ brood) had significantly lower survival than those eclosing in the dry season (1^st^ brood) (*F* = 12.49; df = 1,178; P = 0.02). The forest type*treatment interaction was not significant (*F* = 0.24; df = 3,176; *P* = 0.624), nor was the treatment*brood interaction (*F* = 2.34; df = 3,176; *P* = 0.128), nor the forest*brood interaction (*F* = 0.10; df = 3,176; *P* = 0.757), nor the three-way interaction (*F* = 2.34; df = 7,172; *P* = 0.440).

**Figure 7:**
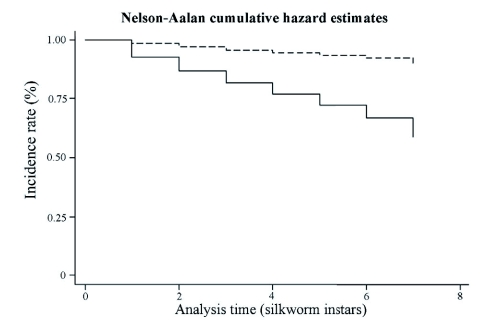
Survival function of *Anaphe panda* silkworms in natural and modified Kakamega Forests when protected with a sleeve net (dashed line) or unprotected (solid line). High quality figures are available online.

The overall survival of silkworm cohorts was significantly higher in the natural forest compared to the modified forest (c2 = 36.6, *P* < 0.001). In the Ischeno modified forest, 38 out of 48 cohorts (79.2%) survived to spin cocoons in the sleeve net treatment, compared to 15 out of 38 (39.5 %) for unprotected controls. In the Ikuywa natural forest 76 of 78 (97.4 %) protected cohorts survived, compared to 51 of 57 (89.5 %) control cohorts. Considering total numbers of larvae, only 2,557 of 15,198 (16.8%) unprotected silkworms survived to pupation in the modified forest, whereas 7,757 out of 12,447 (62.3%) survived when protected with sleeve nets. In the natural forest, 3,511 out of 17,213 (20.4%) unprotected silkworms survived, compared to 8,888 out of 13,124 (67.7%) in the sleeve nets. Thus, protection with sleeve nets increased survival 3.7 and 3.3 fold in the modified and natural forests, respectively. The Nelson-Aalen cumulative hazard function (Figure 7) reveals that the protective environment provided by net sleeves significantly reduced (*F* = 202.04; df = 1,125; *P* < 0.001) silkworm larval mortality across all instars, although mortality was greater in early instars than in later ones.

## Discussion

The ‘force of mortality’ (incidence rate) for each instar can be calculated as follows:

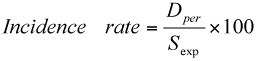

Where *D _per_* = Number of dead larvae in the specific instar and *S _exp_* = Number of surviving larvae exposed to risk in that instar.

### Data analysis

The data on numbers of larvae surviving to form cocoons were analyzed by factorial ANOVA (SAS Institute, 2003) with ‘year’ (2005/2006/2007), ‘treatment’ (protected/unprotected), forest (natural/modified), and ‘brood’ (1^st^/2^nd^) as independent variables. Analysis of survival-time data was carried out with the software Stata7 (STATACORP, 2004) and a Chi-Square test was used to compare overall cohort survival between forest types.

## Results

### Environmental data

Data on mean monthly average temperature, relative humidity, and rainfall for the two study sites are reported in Figures 5 and 6, respectively. Note that rainfall is bimodal in the Kakamega Forest, with a period of “long rains” from April through June, and “short rains” in August through November. During the study, the annual rainfall ranged from 180.9 to 265.9 cm in the modified forest (Isecheno), and from 188.6 to 224.7 cm in the natural forest (Ikuywa). The annual number of rainy days ranged from 196 – 219, and from 207 – 209 in the modified and natural forests, respectively. Mean monthly maximum temperature ranged from 15.5° C to 36.8° C at Isecheno, and from 16.5 – 35.6° C at Ikuywa. Mean monthly maximum humidity ranged from 45.4 – 86.2 % in the modified forest and from 35.6 – 80.9 % in the natural forest.

### Larval survival

There was no effect of ‘year’ on the number of larvae pupating (*F* = 0.09; df = 2,177; *P* = 0.910) so data were pooled across years for further analysis. In both forest types, mortality rates tended to be higher for young larvae (1^st^ to 4^th^ instar) than for older instars (5^th^ to 7^th^) (Table 1), but the main effect of forest type was not significant (*F* = 2.49; df = 1, 178; *P* = 0.117). However, the effect of the sleeve net protection treatment was highly significant (*F* = 229.26; df = 1,178; *P* < 0.001) and cohorts eclosing in the wet season (2^nd^ brood) had significantly lower survival than those eclosing in the dry season (1^st^ brood) (*F* = 12.49; df = 1,178; P = 0.02). The forest type*treatment interaction was not significant (*F* = 0.24; df = 3,176; *P* = 0.624), nor was the treatment*brood interaction (*F* = 2.34; df = 3,176; *P* = 0.128), nor the forest*brood interaction (*F* = 0.10; df = 3,176; *P* = 0.757), nor the three-way interaction (*F* = 2.34; df = 7,172; *P* = 0.440).

The overall survival of silkworm cohorts was significantly higher in the natural forest compared to the modified forest (c2 = 36.6, *P* < 0.001). In the Ischeno modified forest, 38 out of 48 cohorts (79.2%) survived to spin cocoons in the sleeve net treatment, compared to 15 out of 38 (39.5 %) for unprotected controls. In the Ikuywa natural forest 76 of 78 (97.4 %) protected cohorts survived, compared to 51 of 57 (89.5 %) control cohorts. Considering total numbers of larvae, only 2,557 of 15,198 (16.8%) unprotected silkworms survived to pupation in the modified forest, whereas 7,757 out of 12,447 (62.3%) survived when protected with sleeve nets. In the natural forest, 3,511 out of 17,213 (20.4%) unprotected silkworms survived, compared to 8,888 out of 13,124 (67.7%) in the sleeve nets. Thus, protection with sleeve nets increased survival 3.7 and 3.3 fold in the modified and natural forests, respectively. The Nelson-Aalen cumulative hazard function (Figure 7) reveals that the protective environment provided by net sleeves significantly reduced (*F* = 202.04; df = 1,125; *P* < 0.001) silkworm larval mortality across all instars, although mortality was greater in early instars than in later ones.

## Discussion

The climatic conditions observed in this study (Figures 5 & 6) were consistent with reports by Muriuki and Tsingalia (1990) and Kokwaro (1988). As Poikilothermie organisms, the life cycle, activity, distribution and abundance of Lepidoptera are influenced by temperature (Hill et al. 1999). Pollard and Yates (1985) found that temperature and rainfall were likely to influence the survival of butterflies directly and indirectly through the effects on plant growth, disease, predation or other factors. In light of the present study, further work is warranted to understand why a forest insect like *A. panda* periodically develops high populations in certain well-defined types of forest habitat, but not in all habitats where it occurs.

**Table 1:**
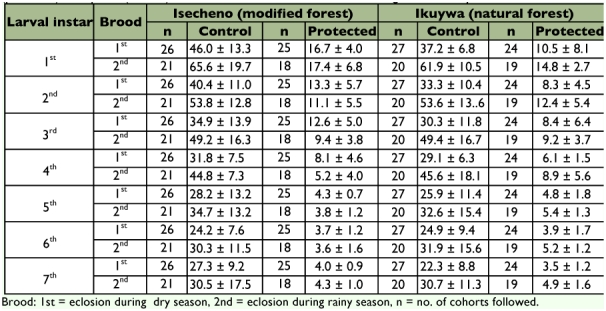
Mean instar-specific mortality (perecent ± SEM) of Anaphe panda silkworms either enclosed in net sleeves (protected) or exposed (control) in modified versus natural tracts in Kakamega Forest, Kenya.

Predation likely contributed to mortality of young larvae, and disease to mortality of older instars. The larval parasitoids *Exorista cardinalis* F. (Diptera: Tachinidae) and *Cryptus leucopygus* Granenhorst (Hymneoptera: Ichneumonidae) were recovered in mummified larvae during the course of the study. These findings are consistent with parasitoids recovered from silkmoths by Kioko et al. (1999) and Raina (2004) and indicate that sleeves nets can be used to reduce silkworm mortality due to natural enemies, especially during the rainy season when larval mortality tends to be higher. This simple technology has the potential to improve the commercial viability and sustainability of the wild silk industry in Africa. The introduction of wild silk production to the Kakamega forest may offer important economic incentives to farmers surrounding the forest. More than 12,400 ha are suitable for silkworm food plants in the Kakamega Forest and could be utilized for the cultivation of *B. micrantha* (Mbahin et al. 2008). Although the use of sleeve nets greatly improved the survival rate of silkworms in both forest habitats, overall cohort survival was somewhat higher in the natural forest than in the modified forest that contained introduced tree species. Thus, reforestation with indigenous species such as *B. micrantha* will not only favor the conservation of indigenous biodiversity, but also enhance the productivity of the wild silkmoth industry.
